# Supplementary figures and images for: A Host-Pathogen Interaction Screen Identifies ada2 as a Mediator of Candida glabrata Defenses Against Reactive Oxygen Species
Source: G3 (Bethesda). 2018 Mar 13;8(5):1637–47. doi: 10.1534/g3.118.200182 (PMC5940155; doi:10.1534/g3.118.200182)

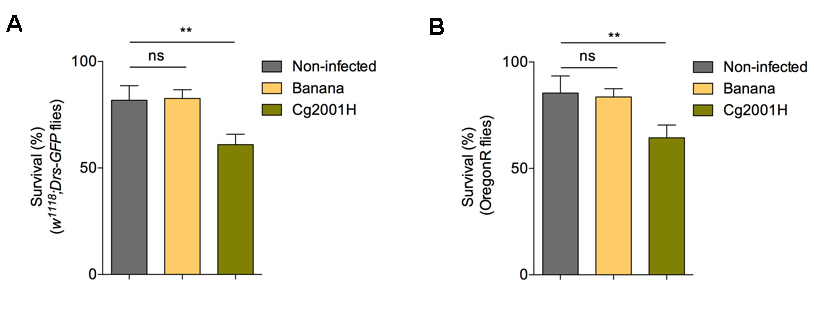

Supplement: Supplementary file 1 [file 1637FigureS1.tif]

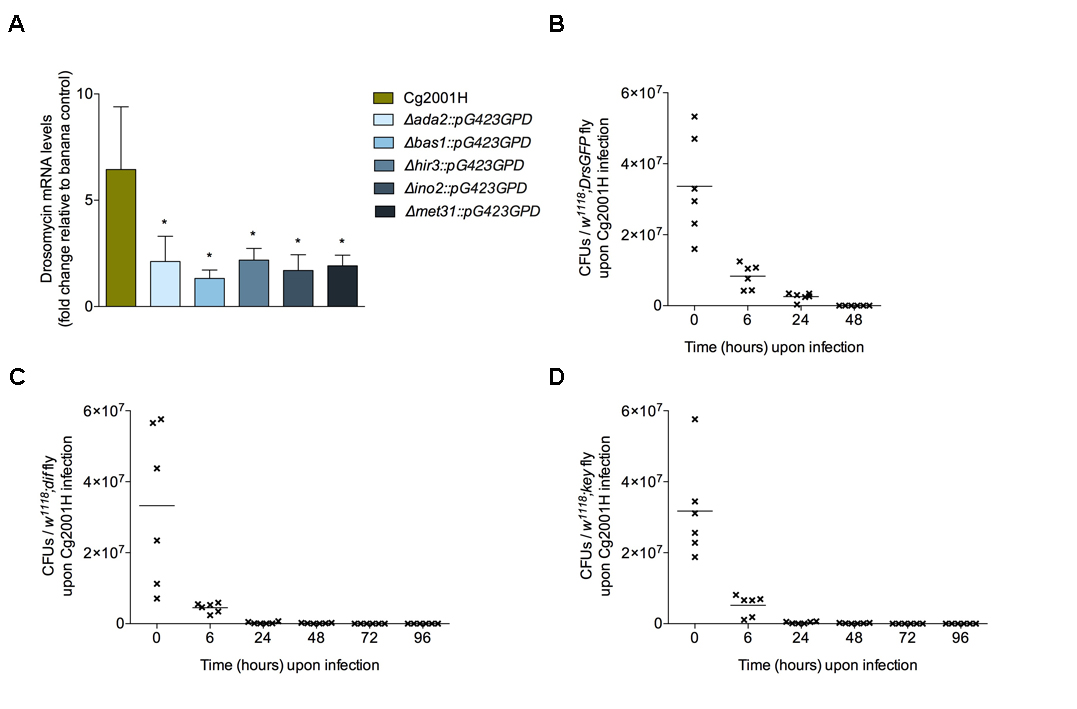

Supplement: Supplementary file 2 [file 1637FigureS2.tif]

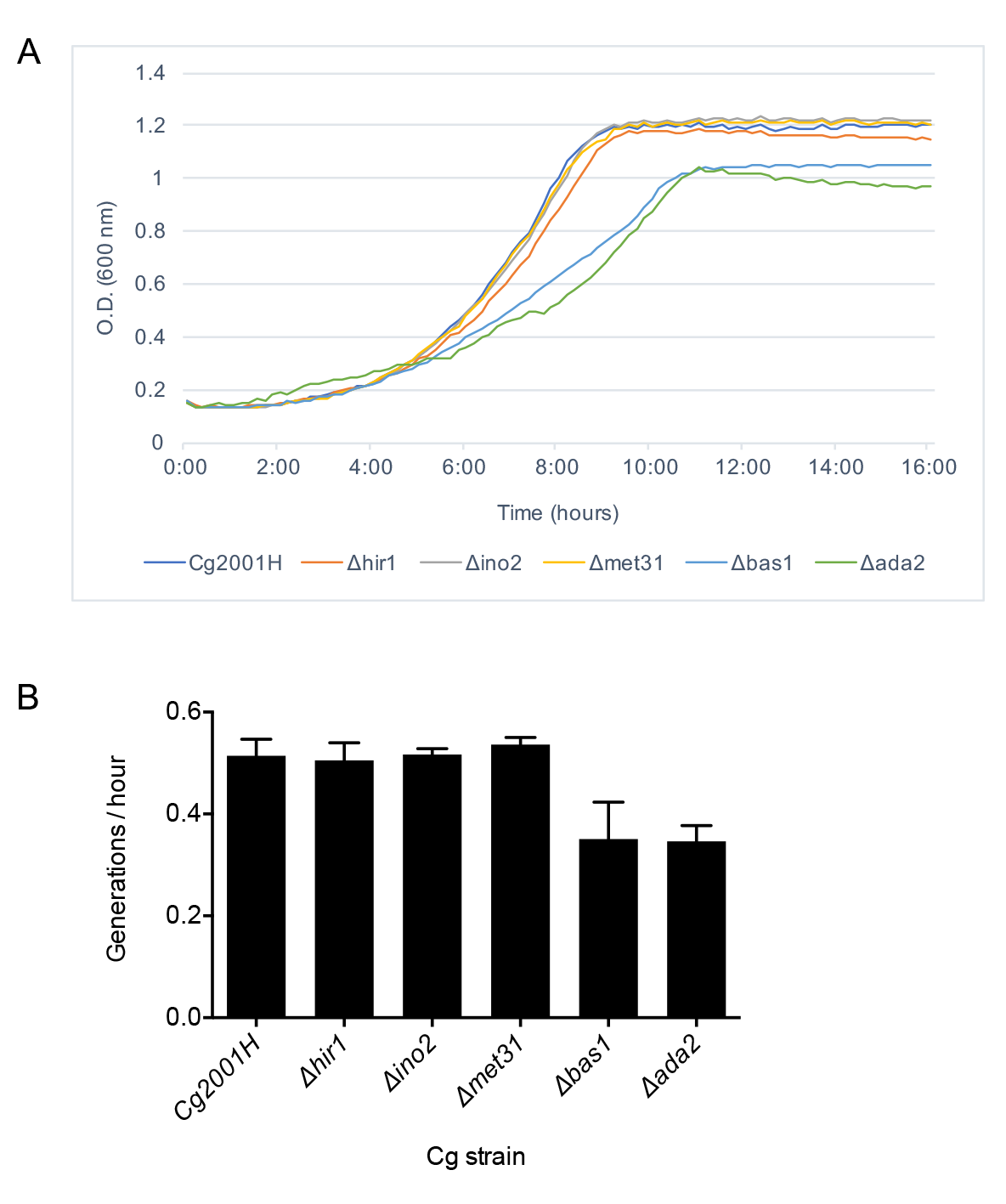

Supplement: Supplementary file 3 [file 1637FigureS3.tif]

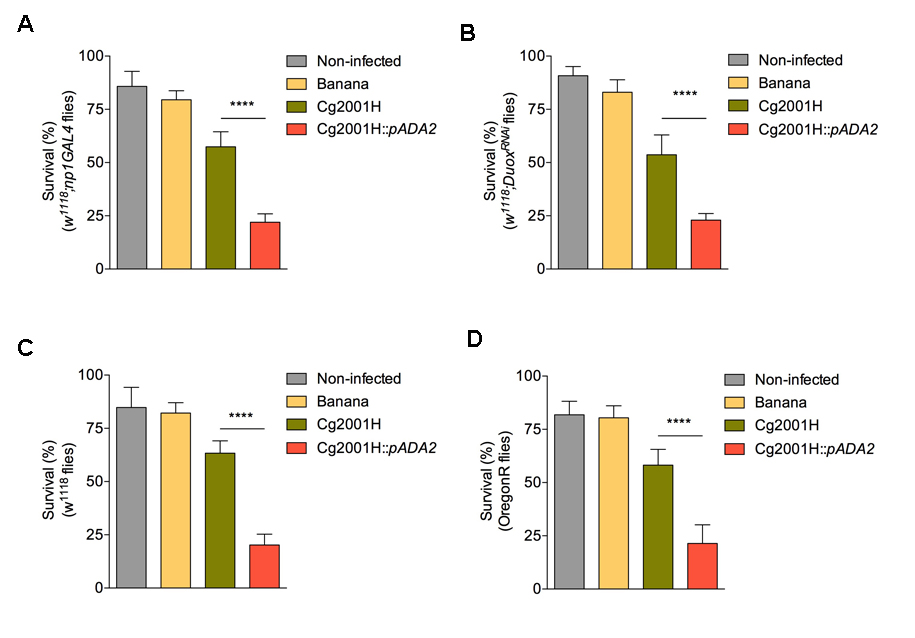

Supplement: Supplementary file 4 [file 1637FigureS4.tif]
